# Postmortem morphology of honeybee stings induced fatal anaphylaxis

**DOI:** 10.1007/s00414-025-03483-5

**Published:** 2025-04-03

**Authors:** Katerina Grusova, Tomas Vojtisek, Rexson Tse, Tomas Kalinka

**Affiliations:** 1 Department of Forensic Medicine, St. Anne’s Faculty Hospital, Tvrdeho 2a, Brno, 602 00 Czech Republic; 2 Department of Forensic Medicine, Faculty of Medicine, Masaryk University, Brno, Czech Republic; 3 Forensic and Scientific Services, Health Support Queensland, Queensland, QLD Australia; 4 School of Medicine and Dentistry, Griffith University, Southport, QLD Australia

**Keywords:** Forensic pathology, Postmortem, Autopsy, Anaphylaxis, Insect, Honey bee

## Abstract

Despite numerous case reports in the literature, high-quality postmortem images of honeybee sting are scant in postmortem literature. We report a case of a fatal anaphylactic death from honeybee stings with detailed high-quality annotated macroscopic and microscopic morphological images. A woman in her mid-60’s was found dead in her garden cabin unexpectedly after mowing the lawn near her beehives in late evening hours. She passed away despite resuscitation efforts. External examination showed multiple honeybee stings on the body. A number of them had a nidus near the center in which a stinger was confirmed by subsequent histology. Postmortem examination and ancillary testing showed features of anaphylaxis.

## Introduction

The diagnosis of bee stings related deaths requires clinical suspicion, postmortem findings, and ancillary tests. In terms of postmortem findings, the macroscopic and microscopic morphology of bee stings are not well documented or described in literature, despite multiple case reports [1–4]. We report a fatal anaphylactic death from honeybee stings with detailed description and high-quality annotated morphological images.

## Case report

A woman in her mid-60s who was found deceased in her garden cabin with an unopened antihistamine (bisulepin) in the vicinity. She had no significant background medical history and had never seen an allergist. She was last seen alive earlier that evening when she was mowing the lawn where the beehives were located. She was subsequently found by a neighbour unconscious in her cabin, and despite resuscitation efforts she passed away.

**Fig. 1:**
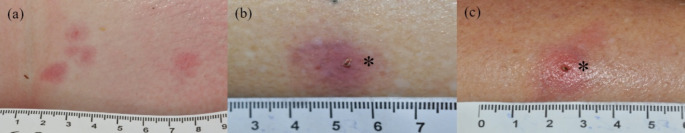
Representative image of one group of the thirty-two cutaneous raised spots with a dimension of 10–15 mm of red-blue colour (**a**), including six of them having a pinpoint black nidus at the centre (*, **b** and **c**)

**Fig. 2:**
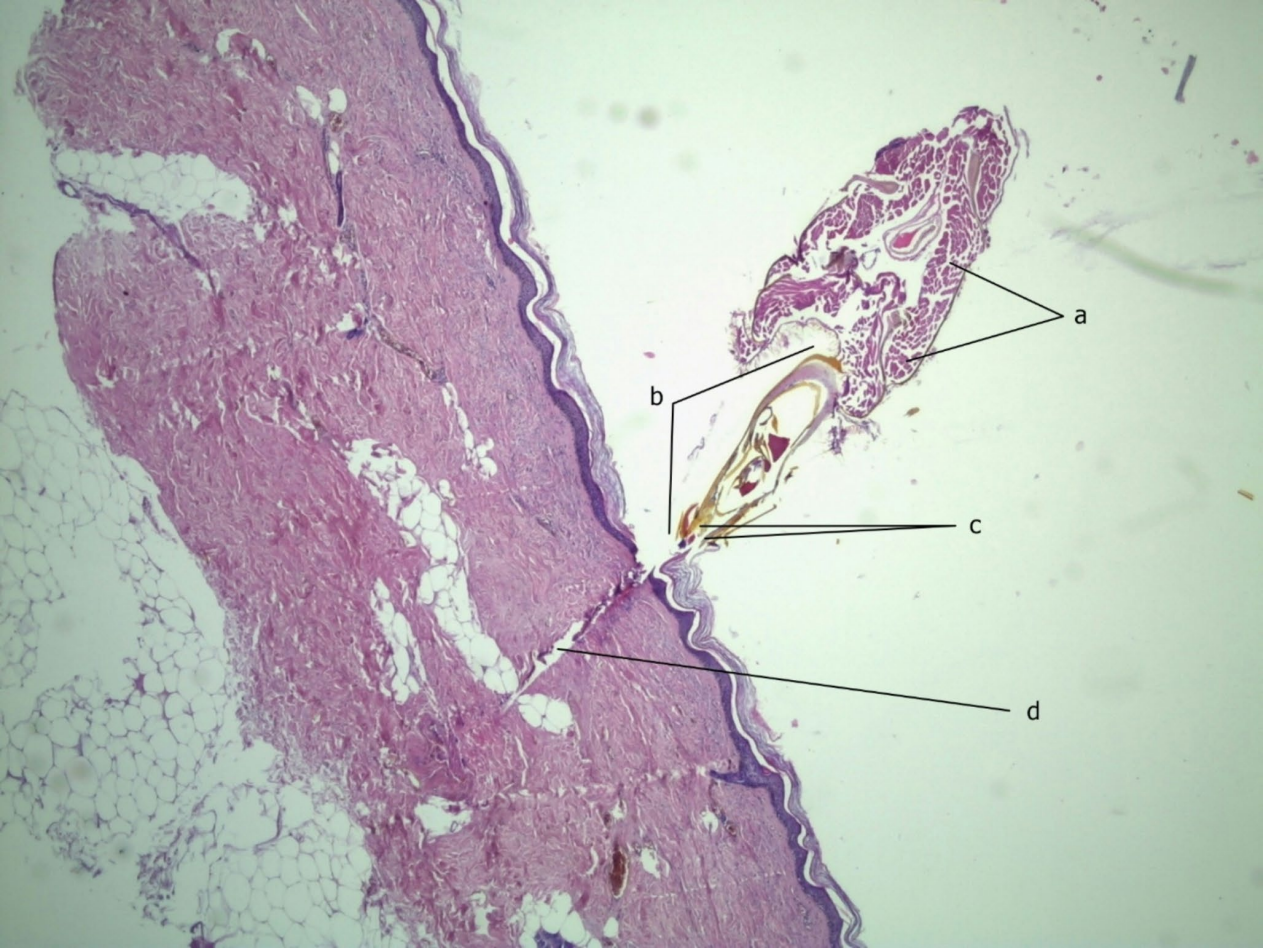
Microscopic image showing the honeybee stinger penetrating the skin with paucity of inflammation and visible structures of the bee’s abdomen. These include the muscle controlling the stinging apparatus (**a**), the sting bulb (**b**), the sting stylet (**c**), and the puncture channel (**d**). The channel would typically contain two sting lancets, but these are likely outside the cutting plane. (hematoxylin and eosin stain, low power)

Postmortem examination was performed 36 h after death at the Department of Forensic Medicine in Brno, Czech Republic. External examination showed approximately thirty-two uniform 10–15 mm round raised red-blue spots distributed on the exposed skin of the torso and limbs, of which six had a nidus near the center, raising the possibility of stingers (Fig. 1). These spots initially raised suspicion of blunt force injury, however, subsequent histological examination of the spots with stingers confirmed a female worker honeybee stinger penetrating the skin with a paucity of surrounding inflammation (Fig. 2). The structures of the female worker honeybee’s abdomen containing the stinging muscle apparatus and sting bulb, together with the sting stylus/lancet with barb and puncture channel were clearly identified on a histological slide. Apart from these spots, the external examination was unremarkable.

**Fig. 3:**
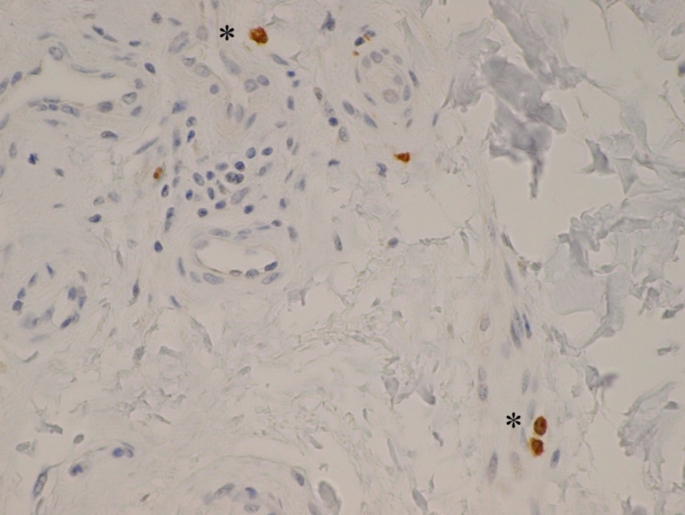
Microscopic image of the larynx (high power) stained with anti-CD117 showing oedema and prominent mast cell (*)

Internal examination showed a range of anaphylactic related changes and histological examination of the larynx showed oedema with prominent mast cells in keeping with anaphylaxis (Fig. 3). No other significant pathology was recognised macroscopically or microscopically. In particular, there was no evidence of envenomation syndrome.

Total tryptase serum levels were significantly elevated at 152ug/L (*N* < 23ug/L, anaphylaxis > 45ug/L) [5–8], total and specific (bee) immunoglobulins E (IgE) were elevated at 153 IU/l (*N* < 100IU/l) and 14.5kU/l (*N* < 0.09kU/l) respectively. Wasp and hornet IgE were slightly elevated at 1.12ku/L (*N* < 0.09kU/l) and 0.17kU/l (*N* < 0.09kU/l). Toxicological analysis was negative.

Based on the circumstances, postmortem findings and ancillary test, cause of death in this woman was fatal anaphylaxis from honeybee stings.

## Discussion

The presented case provides detailed annotated postmortem macroscopic and microscopic morphology of the honeybee sting (female worker bee) with the stinger in situ to the existing postmortem literature.

Identifying insect bite/stings is critical in investigating a suspected death related to insect bite/sting. In terms of honeybee stings, although described in literature, there is a lack of high-quality comprehensive macroscopic and microscopic postmortem images of honeybee stings [1–4]. Case reports and case series in the literature either have a single low power macroscopic or a microscopic image of the honeybee sting site without the stinger. There is also a lack of description of the distribution, and morphological features.

For Czech Republic, the honeybee (*Apis mellifera*) is found alongside with more than 600 species of solitary bees from the superfamily *Apoidea*. Compared to honeybees, solitary bees are neither aggressive nor territorial, and do not pose a danger to humans. The abdomen of a solitary bee is also anatomically different in which some species lack a sting, while in those that do have one, it is usually too weak to penetrate human skin, and their venom is mild. As such a bee sting in this region would be from a honeybee. Macroscopically, honeybee stings occurs on skin exposed areas, and are relatively uniform, raised, and are 10–15 mm diameter, red-blue in colour and occasionally have a nidus at the centre which histological sections may show the stinger.

In terms of stinger, only the female worker and queen honeybee have a stinger which can be differentiated on histology. The female worker honeybee stinger is barbed, whereas the queen has a longer and unbarbed stinger. The barbed stinger is often left behind, along with the venom sac and muscles that continue to pump venom. This ultimately leads to the bee’s death but allows for the morphological identification of a honeybee sting.

## Data Availability

Data sharing not applicable to this article as no datasets were generated or analysed during the current study.
